# Age differences in high frequency phasic heart rate variability and performance response to increased executive function load in three executive function tasks

**DOI:** 10.3389/fpsyg.2014.01470

**Published:** 2015-03-05

**Authors:** Dana L. Byrd, Erin T. Reuther, Joseph P. H. McNamara, Teri L. DeLucca, William K. Berg

**Affiliations:** ^1^Psychology and Sociology, Texas A&M University-KingsvilleKingsville, TX, USA; ^2^Department of Psychiatry, LSU Health Sciences Center-New OrleansNew Orleans, LA, USA; ^3^University of Florida, Gainesville, FLUSA; ^4^Nemours BrightStart, Jacksonville, FLUSA

**Keywords:** planning, inhibition (psychology), working memory, heart rate variability, respiratory sinus arrhythmia, child, adult

## Abstract

The current study examines similarity or disparity of a frontally mediated physiological response of mental effort among multiple executive functioning tasks between children and adults. Task performance and phasic heart rate variability (HRV) were recorded in children (6 to 10 years old) and adults in an examination of age differences in executive functioning skills during periods of increased demand. Executive load levels were varied by increasing the difficulty levels of three executive functioning tasks: inhibition (IN), working memory (WM), and planning/problem solving (PL). Behavioral performance decreased in all tasks with increased executive demand in both children and adults. Adults’ phasic high frequency HRV was suppressed during the management of increased IN and WM load. Children’s phasic HRV was suppressed during the management of moderate WM load. HRV was not suppressed during either children’s or adults’ increasing load during the PL task. High frequency phasic HRV may be most sensitive to executive function tasks that have a time-response pressure, and simply requiring performance on a self-paced task requiring frontal lobe activation may not be enough to generate HRV responsitivity to increasing demand.

## INTRODUCTION

Executive function is an umbrella term used to group a variety of complex cognitive functions that utilize the attentional control unit of Baddeley’s working memory (WM) model which governs allocation of attention and inhibition of automatic or incorrect action. This Central Executive of Baddeley’s model utilizes neural connections within the frontal lobes as part of their neural circuitry (Baddeley, 1996; Banich et al., 2000; Jansma et al., 2000; Newman et al., 2003; Owen et al., 2005). This category of executive functions includes a number of abilities and their related tasks. A latent factor analysis of performance on a large number of executive tasks found both a unity to executive functions, as well as separate categories of executive functions (Miyake et al., 2000). For both adults and children, the separate categories included updating of WM and inhibition of automatic/over-learned responses, as well as shifting of attention and action (Miyake et al., 2000; Huizinga et al., 2006). Another executive function, multistep planning toward a goal, has been found to rely on attentional control (Baddeley, 1996) and frontal lobe functioning (Luria, 1966; Shallice, 1982; Unterrainer et al., 2004a; Kaller et al., 2011).

There is a prolonged child development of neural circuitry that differs for various executive functions shows increases in area growth, efficiency of activity, and myelination including in frontal areas from preschool to late adolescence, as well as increased coordination with age of frontal connections’ coordinated neural functioning as measured by electroencephalographic coherence (e.g., Casey, 1992; Casey et al., 1997; Hanlon et al., 1999; Thomas et al., 1999; Nelson et al., 2000; Durston et al., 2001; Fuster, 2002; Thatcher et al., 2008). As might be expected, this is accompanied by a prolonged development of executive function task performance, with particularly large improvements during preschool/kindergarten and adolescent years (Klahr and Robinson, 1981; Welsh et al., 1991; Diamond and Taylor, 1996; Denot-Ledunois et al., 1998; Zelazo, 2000; Davidson et al., 2006; Lamm et al., 2006; Zelazo and Müller, 2007; Kaller et al., 2008; Unterrainer et al., 2014).

### PHASIC HIGH FREQUENCY HEART RATE VARIABILITY

A psychophysiological measure, phasic high frequency heart rate variability (HRV), may provide valuable information about the modulation of executive control in children and adults. According to Thayer et al. (2009), prior to 1867 Claude Bernard was the first to suggest that cortical activity has a reactive response on heart rate. Since then, it has been found that the heart rate can fluctuate at a wide range of frequencies slow, medium, and fast (Jennings and Yovetich, 1991), with the faster frequency associated with typical inhalation and exhalation rates. Thus, respiratory related HRV has been measured as the spectral power of the heart rate changes within the frequency range of respiration. This measure is somewhat similar to another psychophysiological measure, respiratory sinus arrhythmia (RSA), also measures the synchrony of respiration and heart rate.

Although different terms have been used to define the cognitive process indexed by high frequency HRV, referred to from this point on as HRV, or RSA, there is general agreement that reduced/suppressed RSA or HRV power (less power in the frequency band of respiration) is associated with increased effortful mental processing or effortful attentional control in adults (Porges and Byrne, 1992; Suess et al., 1994; Berntson et al., 1997; Beauchaine, 2001; Hansen et al., 2003; Mulder et al., 2004; Porges, 2007). While it has been concluded that HRV is related to executive task performance and reflects the prefrontal utilization required by active control of attention, there is also a call for further research into which executive functions change HRV (Thayer et al., 2009). This report was limited to examining primarily inhibitory processes.

Empirical evidence supports this link between frontal lobe activation and mental effort during executive function. The role of the frontal cortex in the regulation of HRV has been demonstrated with clinical populations (Althaus et al., 1999, 2004; Lane et al., 2001) as well as functional imaging studies with normative populations (Gianaros et al., 2004; Matthews et al., 2004).

A number of functional imaging – HRV studies have found a relationship between increased activation of the anterior cingulate cortex (ACC) and decreased RSA in frequencies similar to that of respiration (Critchley et al., 2003; Matthews et al., 2004). It has been theorized that the ACC serves to detect instances where it is necessary to recruit frontal areas, including the dorsolateral prefrontal cortex, to manage increasing executive demands (Botvinick et al., 2001; Hajcak et al., 2003).

In a model of the heart–brain connection by Thayer et al. (2009), sympathetic and parasympathetic regulation of HRV is modeled as modulating with increased dorsolateral prefrontal and ACC activation such that increased activation results in decreased HRV. Additionally, part of a model by Thayer et al. (2009) suggest that activation of the prefrontal cortex can result in discontrol of the heart rate response through both a tonic acceleratory drive and a tonic deceleratory drive from both the sympathetic and parasympathetic branches of the autonomic nervous system. We suggest that this results in disregulation of the heart rate response, which we propose would result in decreased phasic high frequency HRV.

Due to their undeveloped frontal neural circuitry, children may be less able or less consistent in their ability to activate the ACC and recruit their underdeveloped frontal areas to manage the difficult executive task conditions, thus deregulating their HRV. That is, children may have less ability to fully recruit the attentional/behavioral control system, including the dorsolateral prefrontal cortex, in order to manage the task conditions. However, this stands in opposition to a model by Thayer et al. (2009) which suggests less activation of the prefrontal cortex would lead to activation of the central nucleus of the amygdala, which would lead to an increase in sympathetic activity and inhibition of the parasympathethoexcitatory neurons, which in turn would lead to a decrease in vagal tone and HRV. It is worth note that this model is based on animal models and adult neurology, and may not apply to the hypofrontality due to a lack of development.

### THE CURRENT STUDY

As compared to HRV during a rest period, decreases in HRV have been found during executive function tasks with both adults (Hansen et al., 2003; Johnsen et al., 2003) and children (Hickey et al., 1995; Mezzacappa et al., 1998). We expect from prior research that children will show less HRV responsitivity during the Stroop task (as seen in a younger and older adult developmental study of a variant of the Stroop task, Mathewson et al., 2010), and perhaps also the Tower of London task that requires inhibition of inefficient moves to make the correct counterintuitive correct moves. Studies of HRV during executive function tasks have not, however, been simultaneously assessed with multiple subtypes of executive functions, especially in children. One study compared HRV during Stroop task and mental arithmetic in older adults. This study found that mentally stimulating activities predicted HF-HRV (Lin et al., 2013). However, this was one formal executive function task, the Stroop, and another cognitively challenging task, which likely requires executive functions such as WM, mental arithmetic. The current study, a study including tasks tapping into the subtypes of executive functions would allow for the comparison of developmental differences, which developmental studies show less HRV response in children and animal models and adult neurology suggest increased HRV. We expect to see both performance on each dimension of executive function and in the HRV changes that are associated with increased executive functioning load in both age groups, but our hypotheses about developmental HRV are exploratory.

In the current study we have the goal to examine our child group for developmental differences compared to adults. We approach these goals using three executive functioning tasks which typify subtypes of executive function (Baddeley, 1996; Miyake et al., 2000): inhibition of an automatic/over-learned response, goal-focused multi-step planning, and WM updating. With these tasks we utilized a parametric study design rather than a baseline rest design. A parametric design allows for the calculation of difference scores to a low level of the task to assess increases or decreases in adults’ and children’s physiological and behavioral responsitivity to increased executive functioning load without confounds possible due to individual or developmental differences in interpretation/processing of a rest baseline. In fact we suggest that a rest baseline may be inappropriate for HRV as it is for other psychophysiological measures such as electroencephalogram and functional magnetic resonance imaging as the baseline of rest requires a form of mental effort, especially in children, as they exhibit attentional and motor control and “tune out” all modalities and inhibit all behavioral responses, which may be a challenge when they are in the novel laboratory environment with electrodes and a respiration band on their bodies.

Functional magnetic resonance imaging suggests that a rest baseline is not a “zero” (Stark and Squire, 2001). A parametric design, rather than a rest baseline, is now becoming standard in functional magnetic resonance imaging, especially in developmental studies (Katsoni et al., 2006). Scores, both correctness and speed, were calculated as difference scores relative to the easiest condition. These difference scores allow for the assessment of the participants’ behavioral and physiological reaction to increased cognitive load while controlling for stimulus and developmental motor events. Though the easiest condition of each task may have some executive load, our parametric design still examines changes in performance and physiological response from a lower level of executive load to higher levels of executive load. HRV responsitivity scores and behavioral responsitivity scores are both calculated similarly to task-rest baseline difference scores but are instead calculated relative to the within-task lowest cognitive demand condition. Our primary hypotheses concerned changes due to task difficulty, and thus these scores reflect the response to the increased task demand.

We examined the executive functions of WM and inhibition as they have been found to be a separate factor (Huizinga et al., 2006) and also planning as it requires the combination of WM and inhibition, as well as longer term goal tactic, and is also crucial task for daily functioning (Luria, 1966). We contrasted adult responses with early elementary school age children’s responses for a number of reasons: (a) early elementary school age is above an age span when resting HRV is increasing (Finley and Nugent, 1995), (b) developmental comparisons with early elementary school age are also similar to past behavioral studies evaluating executive functioning age differences (Luciana and Nelson, 2002; Huizinga et al., 2006), and (c) the age of our sample is before the final adolescent growth spurt in executive functioning abilities that occurs during adolescence (De Luca and Leventer, 2008).

### CLINICAL SIGNIFICANCE OF THE STUDY

The current study makes use of multiple tasks that are used for clinical assessment of executive functioning abilities. It may be helpful clinically to know which of these tap into the form of cognitive effort indexed by HF-HRV, and the neural circuitry that underlies the HF-HRV response. Specifically interesting would be if a cognitive process were to in past literature activate frontal regions, but not elicit a parametric change in HRV with difficulty. The spatial resolution of function MRI (fMRI) is such that it may be that the neural regions underlying HF-HRV are not utilized as may appear on fMRI studies, or it might show that that region is being used but not in the way that modulates HRV. These executive functions are important for a large number of clinical concerns, ranging from judging developmental delay, or deficit with a disorder such as ADHD, to assessing atypical aging, where executive functions may be early to decline. Certainly the current study will suggest if the measures used should be considered equivalent when administered clinically to children and adults. They may not if children’s and adults’ HF-HRV responds differently to increased executive demands.

### HYPOTHESES

We hypothesize that, in all tasks, incremental increases in executive functioning load will result in both adults and children presenting incremental decreases in HRV power and behavioral performance. Children may be less able to manage increased executive loads because of their undeveloped frontal control and may, therefore, have smaller changes in quality of performance with increasing executive load. This underdeveloped frontal control may also lead to children’s HRV being less controlled and efficient, with their responsitivity being less incrementally locked to increases in executive load. Whether children’s HRV will be higher or lower than adults is exploratory.

## MATERIALS AND METHODS

### PARTICIPANTS

Data were analyzed from 25 children (16 male, 6–10 years, *M* age = 8.6 years) from local schools and 34 adults (19 male, 18–25 years, *M* age = 22.0 years) from introductory psychology courses. Child participants were recruited through flyers posted at graduate student on-campus housing, since this housing is often utilized by graduate students with children. Adult participants were recruited through an undergraduate psychology subject pool. Adult and child samples included participants who, according to a self/guardian report questionnaire, were in good present and past health and currently taking no medications. All participants were recruited and tested using procedures in accordance with the Ethical Guidelines of Psychologists and Code of Conduct of the American Psychological Association (1992) and approved by the university Institutional Review Board.

### DESIGN AND PROCEDURE

Upon arrival at the university laboratory, adult participants or child–parent pairs heard a brief description of the study and underwent consent/assent procedures. Adult participants or child–parent pairs then answered a questionnaire about the participants’ basic demographic data, current and past health, medical/psychiatric diagnoses, and medications the participants were currently taking. The experimental session (∼45 min) then began.

The experimental session consisted of the researcher briefing the participants about the tasks, referred to as “puzzle games,” and the opportunity to earn a performance bonus of up to $5. This bonus was in addition to the standard compensation of $5 for children and class credit for adults. All participants were encouraged equally but were not informed about their progress toward performance bonuses until the end of the experiment. Encouragement and financial incentive were used to address potential decreased vigilance, engagement, and/or effort across the session, which is a major concern when testing child participants. Use of financial incentive was particularly crucial in the current study due to the past research finding that young school age boys’ performance and HRV revealed more attention to task when the children were offered monetary reward (Suess et al., 1997).

The experimenter escorted the participants to a sound-attenuated booth and fitted the participants with electrocardiogram (ECG) electrodes and a respiration gage belt. Participants were instructed to refrain from speaking and making non-task-related movements during data-collection/task periods. Participants then began the three computerized executive function tasks, the Day/Night Stroop, the Tower of London, and the N-Back, with the order of the tasks determined by a Latin square, counterbalanced design. There was no significant evidence that child or adult participant groups performed more poorly on tasks later in the session ^1^. Difficulty conditions within each task were completed in order of increasing executive load levels to reduce discouragement, a concern especially in children.

Before each difficulty condition, there was an experimenter-participant interactive break during which the experimenter provided encouragement and instructions. Instructions using a standard script and pictures, either in a flip-book or on a computer screen, consisted of the experimenter explaining the stimuli, responses, and objectives for the next level of difficulty. Next, the experimenter demonstrated the difficulty condition and the participants were given two opportunities to practice this difficulty condition. The experimenter corrected and guided the participants if they performed incorrectly during the practice opportunities. If participants’ responses on the practice periods revealed a lack of understanding of the condition, the experimenter repeated the instructions, example, and practice session. Only two children required re-instruction, both on the N-Back task.

### EXECUTIVE FUNCTIONING TASKS

Our tasks were designed to: (a) be appropriate for both children and adults, (b) tap into the cognitive function of interest across a range of difficulty, and (c) be free of either periodic stimuli or large motor movements that could modify the participants’ heart rate patterns. Tasks were identical for children and adults. In all tasks conditions were presented in periods of 3-min each so as to equate temporal conditions in evaluating the HRV.

#### Inhibition task (Day-Night Stroop)

The task employed was a variant of the standard color-word Stroop (1935) task, which is widely used to measure response inhibition in adults. Although letters are easily recognized by children as young as 6 years of age, reading automaticity is achieved later in development (Saint-Aubin et al., 2005). This lack of reading automaticity makes the color-word version of the Stroop task less valid for children. Although picture-based Stroop variants, including the Day/Night Stroop task we employed, are more common in developmental research, they are effective at eliciting difficulty in automatic response inhibition from adults as well as children. This is evident as slower response times in the stimulus–response conflict condition (Diamond and Taylor, 1996; Diamond and Kirkham, 2005; Davidson et al., 2006).

In the most common administration of the Day/Night Stroop (Gerstadt et al., 1994) participants speak either matching (“day” to a picture of day) or opposite (“day” to a picture of night) responses to simple, colorful drawings. Our computerized version of this task required only a mouse click to make their picture selection and this allowed for the recording of respiration and HRV without contamination from speech-related artifact and allowing us millisecond response accuracy; otherwise it was very similar to the spoken Day/Night Stroop. Participants were presented with a sequence of images, cartoons of either day or night, appearing one at a time in the upper portion of the computer screen. In the lower portion of the screen were two smaller images, one of day and one of night, which served as response buttons when left clicked. Participants used a computer mouse to click on the matching picture in the control difficulty condition and the opposite picture in the inhibition difficulty condition. Following the response, the next picture in the series of images appeared in 500–2000 ms, with the inter-stimulus-intervals independently randomized for each participant.

During the inter-stimulus interval, participants moved their mouse cursor to a bulls-eye image located between the two response images. This prevented anticipatory movements and held constant the movement distance for each response button. The matching (control) difficulty condition aways preceded the mismatching (response inhibition) difficulty condition, with each condition period being 3 min. The instructions for both difficulty conditions emphasized responding both quickly and correctly.

#### Planning task (Tower of London)

The Tower of London is a task used clinically and experimentally with both children and adults to measure multi-step planning (Shallice, 1982; Krikorian et al., 1994; Anderson et al., 1996; Berg and Byrd, 2002; Berg et al., 2006, 2010). The original task apparatus consisted of three balls, red, blue, and green, placed on three pegs which can hold one, two, or three balls, respectively. The task objective is to transform an initial ball arrangement to match a goal ball arrangement in as few single-ball movements as possible.

In our computerized version of the task, the initial arrangement appeared as a large image at the bottom of the screen, and the goal arrangement appeared as a small image at the top of the screen. The minimum number of moves necessary to reach the goal position appeared in a box on the far right of the screen. Participants could begin solving problems as soon as they appeared, though they were encouraged to solve the problem within the minimum number of moves but to continue working on a problem until it was solved, even when they made more than the minimum number of moves required. We chose this administration in order to allow for detailed examination of performance for planfulness (Berg et al., 2006).

To move each ball from peg to peg, the participants made a small hand movement, a drag and drop motion with the computer mouse. When the goal arrangement was reached, the participants clicked a button labeled “Done,” which appeared in the top right corner of the screen. The computer program prevented participants from breaking the rules, placing balls off of pegs or placing too many balls on a peg. Participants were presented three increasing planning load difficulty conditions of the Tower of London – problems requiring a minimum of 4, 5, and 6 moves for an optimal solution. In each difficulty condition, participants continued to solve problems with no maximum of that difficulty level until the difficulty condition period of 3 min was complete. These problems were selected based on minimum number of moves required to solve most efficiently, which is a strong predictor of difficulty (Berg et al., 2010). Unfortunately these data were collected before problem selection became based on other problem parameters such as goal and end start position or subgoals (Kaller et al., 2004). These problem aspects not being controlled may have contributed noise to our difficulty levels.

#### Working memory task (N-Back)

In order to examine participants’ responsitivity to increasing WM load, participants performed four increasingly difficult conditions of the N-Back task. Various versions of this task are commonly used as a measure of WM updating both with adults (e.g., Gevins et al., 1997; McEvoy et al., 1998; Müller et al., 2002) and children (Nelson et al., 2000; Vuontela et al., 2003, 2009; Astley et al., 2009). It is feasible for children 6 years and older to perform this letter-based memory task due to their ability to recognize individual letters (Christian et al., 2000). In the current study, practice trials demonstrated that all child participants were able to recognize the stimulus letters.

In all difficulty conditions, participants viewed a light blue computer screen with a sequence of black upper and lower case letters appearing one at a time in the middle of the screen. There were 51 stimuli in each difficulty condition, one third of which were targets. The stimulus duration was 500 ms and the inter-stimulus-intervals varied from 300 to 1600 ms. Both inter-stimulus interval and target position were randomized independently for each participant. In all difficulty conditions, the participants were instructed that, following each stimulus, they were to press one of two computer keyboard keys: either a green key with their left index finger for a target or a red key with their right index finger for a non-target. Participants were instructed that they should respond to every single stimulus with their best answer, even if they were uncertain. Correctness, not speed of responses, was emphasized, though participants were told that non-responses would be considered incorrect.

Participants performed four difficulty conditions requiring incrementally more WM load: 0-, 1-, 2-, and 3-back difficulty conditions, in that order. The definition of a target stimulus differed by difficulty condition. In the 0-back difficulty condition a target was a single letter presented before the response stimuli for that difficulty condition. For all other difficulty conditions, the participants referred back to their memory of the prior stimuli in order to determine whether or not the current stimulus letter was a target. For these difficulty conditions, a target was always the matching letter, and the letter’s case was to be ignored. The position to check for this match was the letter 1, 2, or 3 positions back in the sequence depending on difficulty condition being tested, 1-, 2-, or 3-back, respectively. Each difficulty condition period lasted 3 min.

### HRV RECORDING AND MEASUREMENT CALCULATION

During each 3-min difficulty condition period, heart rate and respiration were recorded as six consecutive, 30-s epochs. This epoch duration was chosen because it was appropriate for examining frequencies of interest, brief enough to lessen concerns about heart rate non-stationarities (Berntson et al., 1997), and identical to that used by earlier studies of children’s HRV (Suess et al., 1997; Porges et al., 2007).

Electrocardiography was recorded using three 1 cm Ag/AgCl electrodes filled with Microlyte electrolyte gel and secured to the cleaned and lightly abraded skin (Nu-Prep gel) via an adhesive electrode collar. Electrodes were placed in a modified type II arrangement, with two active leads, one on the left ankle and the other on the right collarbone, and a ground lead on the mastoid bone behind the left ear. Pilot testing determined that this placement allowed for unobtrusive electrode application as well as a clear EKG signal with sharply peaked R waves.

The EKG signal was amplified 1000x with a Coulbourn S75-01 bioamplifier, then band pass filtered from 8 to 40 Hz in order to minimize drift, movement artifact, and 60 Hz noise. A custom-designed peak detector was used to find the peak of the R-waves and transform the peak trigger to a short TTL pulse. R-R intervals (time between R-wave-triggered TTL pulses) were recorded with 1 ms accuracy by a custom program.

R-R interval timings were processed oﬄine. All R-R interval editing and checking was conducted by trained and reliable research assistants who edited data unaware of the participants’ task orders. A custom program was used to display the sequence of R-R intervals and edit artifactual intervals (dividing combined R-R intervals or combining R-R intervals interrupted by false triggering of the peak detector). Corrected data were re-checked for errors. Specific care was taken in the editing of R-R interval artifacts due to the large impact even a single artifactual R-R interval can have on the outcome of HRV calculations (Berntson and Stowell, 1998).

Six 30-s epochs were recorded for each difficulty condition of each task. Uneditable and/or unusable heart rate epochs were extremely rare. Three children and one adult had R-R recording artifacts that could not be clearly, reliably edited, resulting in one or more unusable 30-s epochs of data. Overall, 99.6% of the children’s data and 99.8% of the adults’ data were included in the analyses.

Using a custom BASIC program, the corrected series of R-R intervals during each 30-s epoch was re-sampled into 250-ms bins. This transformed the R-R intervals into a time-based sequence of R-R interval data, a series of densely sampled weighted R-R intervals for each 250 ms during the 30 s epoch. Further oﬄine processing of R-R interval samples was conducted using Microsoft Excel. Linear trends were removed from each epoch’s time-based sequence of R-R intervals using a linear regression model. Each epoch’s de-trended time series was subjected to a fast Fourier transform (FFT) to obtain the power present in the different spectral bands. HRV values for each difficulty condition were calculated by taking the natural log of each 30-s epoch’s absolute power in the frequency band associated with respiration, then averaging together these natural logs across the six 30-s epochs recorded during each difficulty condition.

Slightly different respiration frequencies were examined for child and adult groups, 0.15–1.03 Hz for adults (a frequency band common to adult studies of HRV and RSA; see Berntson et al., 1997) and 0.28–1.03 Hz for children (a frequency band similar to the frequency band 0.24–1.04 Hz common to child studies of HRV and RSA; see Hickey et al., 1995; Suess et al., 1997; Porges et al., 2007). These frequencies were empirically confirmed from respiration recordings taken during the current study ^2^.

### BEHAVIORAL PERFORMANCE RECORDING AND MEASUREMENT CALCULATION

A total of six behavioral measures were analyzed, two behavioral measures for each of the three tasks, a measure of correctness and a measure of speed (as suggested by Berg and Byrd, 2002). The behavioral measures calculated to capture Day-Night Stroop task performance were the proportion of correct responses given and the response time for all responses. The performance measures for the Tower of London were the number of perfect solutions (solved in the minimum moves possible) and the time taken to solve Tower of London problems (from first move to last). The N-Back performance measures were the proportion of correct responses and response time for all responses.

## RESULTS

### PRELIMINARY DATA PROCESSING

Raw behavioral performance variables were analyzed for strong skew. Variables where the absolute value of the mean of the Fisher kurtosis score divided by the standard error was two or larger (*z*skew = | Skew|*/SE*skew) were transformed using the natural log (Royston, 1992). All behavioral scores, excluding the Stroop proportion correct responses and Tower of London number perfect solutions, were transformed.

The few missing scores, 2% of the data, resulted from random, non subject-specific causes (e.g., computer error during testing, uneditable data, corrupted computer file). In order to maintain the sample size across tasks, missing scores were estimated using the expectation–maximization (EM) method (Dempster et al., 1977; Little and Rubin, 1987).

All measures, including correctness, response speed, and HRV, were then converted into “responsitivity scores” to test directly our hypotheses about the developmental responsitivity to increased executive load in performance and HRV. For the Day/Night Stroop task, responsitivity was calculated as the inhibition (opposite) difficulty condition relative to the control (matching) difficulty condition. For the Tower of London task, responsitivity in the 5-move and 6-move difficulty conditions was examined relative to the 4-move difficulty condition. For the N-Back task, responsitivity in the 1-, 2-, and 3-back difficulty conditions were examined relative to the 0-back difficulty condition. All raw scores and transformed responsitivity scores are presented in **Table 1**.

**Table 1:** Descriptive statistics for raw and reactivity/responsitivity performance and HRV measures for each task.

			Raw scores				Skew-corrected reactivity scores			
			Children		Adults		Children		Adults	
Task	Dependent variable	Difficulty	*M*	*SE*	*M*	*SE*	*M*	*SE*	*M*	*SE*
Stroop	Proportion correct	Control	1	0.001	0.99	0.003	–	–	–	–
		Inhibition	0.99^c^	0.002	0.97^c^	0.006	-9.458^a^	1.080	-5.500^a^	0.662
	Response time (ms)	Control	929.188	32.702	590.305	10.779	–	–	–	–
		Inhibition	1216.541	59.675	703.421	16.89	0.114	0.011	0.075	0.005
	HRV	Control	7.844	0.109	7.781	0.068	–	–	–	–
		Inhibition	7.798	0.115	7.674	0.07	-0.046^b^	0.034	-0.107	0.034
Tower of London	Num perfect solutions	4-Move	4.826	0.513	8.411	0.544	–	–	–	–
		5-Move	2.522	0.301	4.382	0.437	-0.177^a^	0.060	-0.344^a^	0.053
		6-Move	0.957	0.204	2.5	0.373	-0.452^a^	0.058	-0.573^a^	0.050
	Time to solve (sec)	4-Move	23.42	3.137	17.557	0.981	–	–	–	–
		5-Move	43.631	8.033	38.396	3.153	0.223	0.07	0.314	0.03
		6-Move	41.969	5.848	66.503	7.574	0.239	0.061	0.508	0.046
	HRV	4-Move	7.901	0.117	7.712	0.064	–	–	–	–
		5-Move	7.889	0.126	7.751	0.062	-0.012^b^	0.036	0.039^b^	0.026
		6-Move	7.872	0.134	7.722	0.063	-0.029^b^	0.043	0.010^b^	0.035
N-Back	Proportion correct	0-Back	0.861	0.022	0.976	0.005	–	–	–	–
		1-Back	0.78	0.024	0.944	0.007	-0.016	-0.005	-0.007	0.001
		2-Back	0.666	0.026	0.915	0.011	-0.038	0.005	-0.013	0.002
		3-Back	0.63	0.017	0.819	0.014	-0.038	0.004	-0.024	0.003
	Response time (ms)	0-Back	627.146	26.899	546.938	23.519	–	–	–	–
		1-Back	729.108	46.76	633.063	26.405	0.055	0.014	0.064	0.009
		2-Back	757.084	53.034	798.115	40.769	0.066	0.023	0.157	0.017
		3-Back	684.001	45.601	866.159	40.767	0.025	0.021	0.194	0.016
	HRV	0-Back	8.099	0.116	7.768	0.074	–	–	–	–
		1-Back	8.034	0.123	7.791	0.071	-0.064	0.052	0.023	0.035
		2-Back	7.984	0.113	7.688	0.062	-0.114	0.05	-0.08	0.044
		3-Back	8.053	0.117	7.609	0.062	-0.046	0.054	-0.159	0.041

*^a^Reaction score not skew-corrected since not necessary.*

*^b^Value not significantly different than baseline value of 0 (single sample *t-*tests conducted for each age group, *p*s < 0.05 relative to baseline).*

*^c^Value not significantly different than ceiling value of 1.0 (single sample *t-*tests conducted for each age group, *p*s < 0.05 relative to maximum score).*

All scores were evaluated for possible ceiling and floor effects as reported below and noted in **Table 1** when significant. Gender differences were examined separately for children and adults using between-subject two-tailed *t*-tests. Only two significant gender differences were found: adult females were more reactive in their n-back, 1-back condition proportion correct (solving a smaller proportion correct relative to 0-back than males) and child females were more reactive in their n-back, 2-back condition HRV (more suppression of HRV). Genders were combined for all analyses except these two measures, which were also analyzed for potential gender interactions.

In order to determine if there were significant age differences within our dependent variables, we conducted a median split of our child group based on age [*N* = 12 younger: *M*(*SE*) = 7.69 (0.23), *N* = 13 older *M*(*SE*) = 9.61(0.12)]. Two of these one-tailed *t*-tests were significantly different. Younger children differed in responsitivity for 2-back and 3-back conditions of the N-back [*t*s(23) > 2.05, *p* < 0.029]. Younger children also showed fewer perfect solutions on the Tower of London than older children [*t*(23) = 2.04, *p* = 0.024]. For these measures a solutions Age Group × Condition analyses will be conducted.

All analyses with repeated measures were Greenhouse–Geisser corrected. When age differences were *a priori* hypothesized, analyses were conducted using single tail *t*-tests.

### AGE AND LOAD DIFFERENCES ON PERFORMANCE AND HRV RESPONSITIVITY

#### Inhibition of automatic response (Day-Night Stroop task)

This task had one responsitivity level due to the task design of a single condition of increased inhibition load (mismatching condition) compared to the condition of no inhibition load (matching condition). For the dependent variable of Stroop proportion of correct responses, both children and adults performed at ceiling, above 0.97 correct responses. Further analysis of this ceiling-level measure was not performed.

A 1-way (Age) analysis of variance (ANOVA) was conducted for the Stroop dependent variable of reaction time. For reaction, time children were more reactive than adults in their slowing of responses to the inhibition condition [*F*(1,59) = 11.50, *p* = 0.001, partial η^2^ = 0.168]. For HRV, means suggested the adults’ responses were somewhat more suppressed during the inhibition condition than those of the children, but this age difference was not significant [*F*(1,59) = 1.51, *p* = 0.225, partial η^2^ = 0.026]. As indicated in **Table 1**, children’s, but not adults’, HRV responsitivity in the inhibition condition was significantly suppressed relative to the control condition [*t*(24) = 1.35, *p* = 0.042].

#### Multistep planning (Tower of London task)

This task had two levels of responsitivity (two levels of increased difficulty) due to the task design of 5- and 6-move conditions each being compared relative to the easiest, 4-move condition. Children and adults were compared in their responsitivity of the number of perfect solutions with increased planning load using an Age × Difficulty Condition (2 × 2) ANOVA. Though the means suggested that adults had a higher number of perfect solutions, the main effect for age was not significant [*F*(1,57) = 1.94, *p* = 0.169, partial η^2^ = 0.033]. Across age groups, the number of perfect solutions decreased from 5-move to 6-move problems [main effect planning load: *F*(1,57) = 17.90, *p* < 0.001, partial η^2^ = 0.239], but this decrease did not differ between children and adults [Age × Planning Load interaction: *F* < 1].

Since analyses presented above indicated a significant age difference within the child group for this measure of 6-move number of perfect solutions, each child subgroup is separately compared to the adult group. The younger child group was significantly different in suppression in number perfect solutions compared to adults [*t*(16) = 2.08, *p* = 0.026]. The older children were not significantly different in suppression of number of perfect solutions compared to adults [*t* < 1].

**FIGURE 1:**
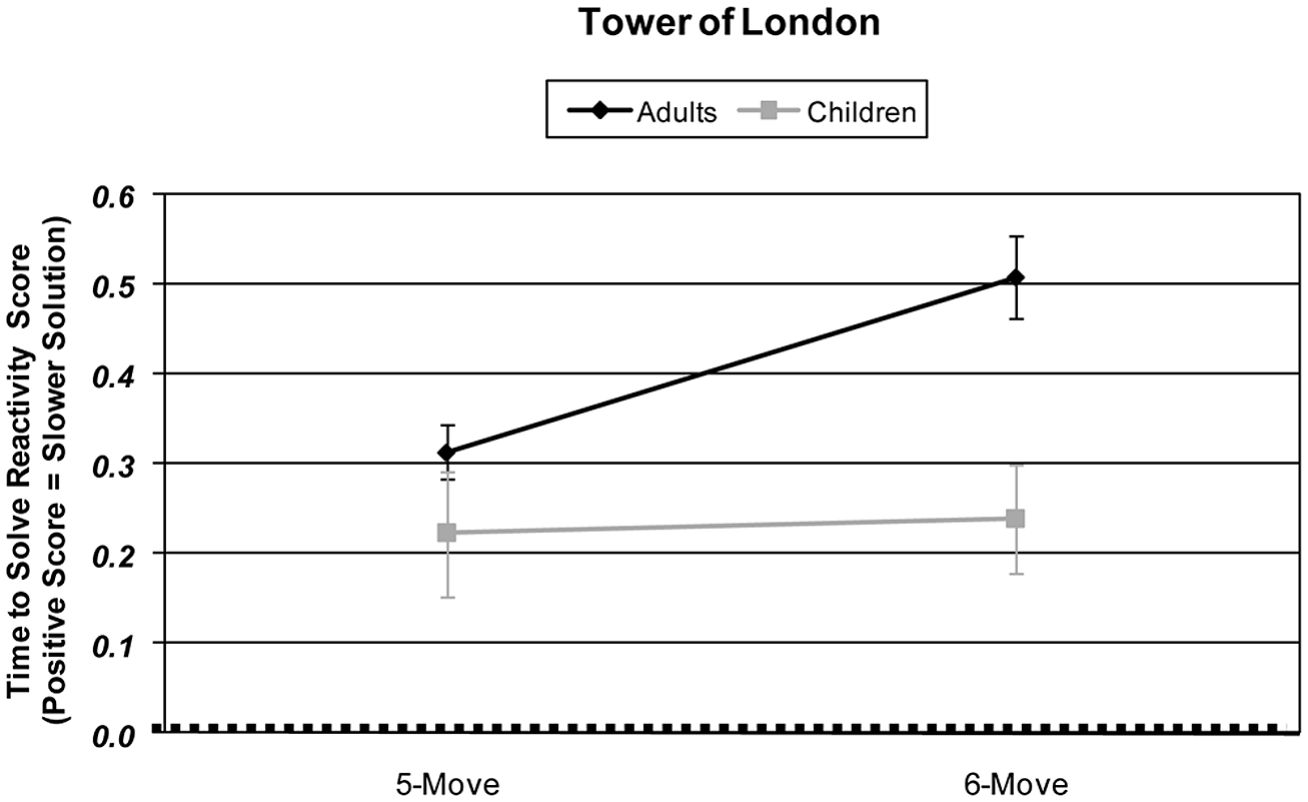
**Age and planning-load-level differences in Tower of London skew-corrected behavioral performance responsitivity as measured by time taken to solve problems (sec).** Responsitivity for each difficulty level is calculated by subtracting that difficulty condition’s performance from the 4-move difficulty condition performance.

Children and adults were compared in their responsitivity of slowing of solution time with increased planning load using an Age × Difficulty Condition (2 × 2) ANOVA. A significant main effect of age revealed that adults’ speed of solution was more reactively slowed than children’s [*F*(1,57) = 7.95, *p* = 0.007, partial η^2^ = 0.122]. A significant main effect of difficulty revealed that participants’ responsitivity in speed of solution was slower with increased planning load [*F*(1,57) = 9.39, *p* = 0.003, partial η^2^ = 0.141]. Children and adults differed in their solution time responsitivity with increased planning load [Age × Difficulty Condition interaction: *F*(1,57) = 6.71, *p* = 0.012, partial η^2^ = 0.105]. When age groups were tested separately, adults exhibited significantly slowed solution time from 5-move to 6-move problems [*t*(33) = 5.19, *p* < 0.001], but children did not [*t* < 1]. See **Figure 1**.

The Age × Difficulty Condition (2 × 2) ANOVA examining HRV found that HRV was not significantly reactive in its suppression with increased planning load [main effect of difficulty condition: *F*(1,57) = 1.37, *p* = 0.247, partial η^2^ = 0.023]. As planning load increased, neither children nor adults changed their HRV suppression [main effect of age: *F* < 1], nor did age group and difficulty condition significantly interact on this measure [Age × Difficulty Condition interaction: *F* < 1]. When age groups were tested independently, neither adults’ nor children’s HRV values were significantly different than baseline [adults: *t*s(33) < 1.37, *p*s > 0.179; children: *t*s(24) < 1].

#### Working memory (N-Back task)

Floor effects for the proportion correct raw scores were assessed by comparing results to the chance performance level of 0.50, the result if target or non-target buttons were randomly pressed. For both age groups, performance in all conditions was significantly better than chance (*p*s < 0.001). Though adults’ proportion correct was high for the 0- and 1-back conditions, their proportions correct were significantly below the ceiling value of 1.00 [*t*s(33) > 4.56, *p*s < 0.001].

Children’s and adults’ proportion correct responsitivity was examined across 1-, 2-, and 3-back conditions using an Age × Difficulty Condition (2 × 3) ANOVA. Children’s decrease in performance was larger than adults [main effect of age: *F*(1,57) = 21.41, *p* < 0.001, partial η^2^ = 0.273]. For both age groups, there was a decrease in proportion correct with increasing WM load [main effect of difficulty condition: *F*(2,114) = 30.41, *p* < 0.001, partial η^2^ = 0.348]. The pattern of proportion correct responsitivity differed for children and adults [Age × Difficulty condition interaction: *F*(2,114) = 5.40, *p* = 0.008, partial η^2^ = 0.081]. Adults’ proportion correct decreased with each level of difficulty [*t*s(33) > 2.38, *p*s < 0.03]. Children’s proportion correct decreased from 1- to 2-back and 1- to 3-back [*t*s(24) > 4.38, *p*s < 0.001], but did not differ from 2- to 3-back [*t* < 1]. The children’s within-group variability was larger than that of the adults.

Children’s and adults’ response time responsitivity was examined across 1-, 2-, and 3-back conditions using an Age × Difficulty Condition (2 × 3) ANOVA. Adults’ responsitivity was more slowed than children’s [main effect of age: *F*(1,57) = 18.65, *p* < 0.001, partial η^2^ = 0.247] and response time responsitivity differed among difficulty conditions [main effect of difficulty condition: *F*(2,114) = 14.96, *p* < 0.001, partial η2 = 0.208]. For the participants as a whole’ response speed responsitivity differed in response to increased WM difficulty conditions [Age × Difficulty Condition interaction: *F*(2,114) = 28.24, *p* < 0.001, partial η^2^ = 0.311]. Adults’ response time responsitivity slowed with each increase in WM difficulty, all pair-wise comparisons were significant [*t*s(33) > 2.68, *p* < 0.011], and each of these adult responsitivity scores was significantly slowed relative to control [*t*s(33) > 7.19, *p*s < 0.001].

Children’s responsitivity in reaction time did not slow from 1- to 2-back [*t*(24) < 1], and their reaction time responsitivity was actually significantly *faster* in 3-back than 2-back [*t*(24) = 3.19, *p* = 0.004]. This pattern resulted in the 3-back reaction time responsitivity nearing significance in its difference from 1-back condition responsitivity [*t*(24) = 2.01, *p* = 0.055]. Children’s response time responsitivity was slower than baseline in the 1- and 2- back conditions [*t*s(24) > 2.81, *p*s < 0.011], but not so in the 3-back condition [*t*(24) = 1.16, *p* = 0.128]. There was generally larger within-group variability in the child data.

**FIGURE 2:**
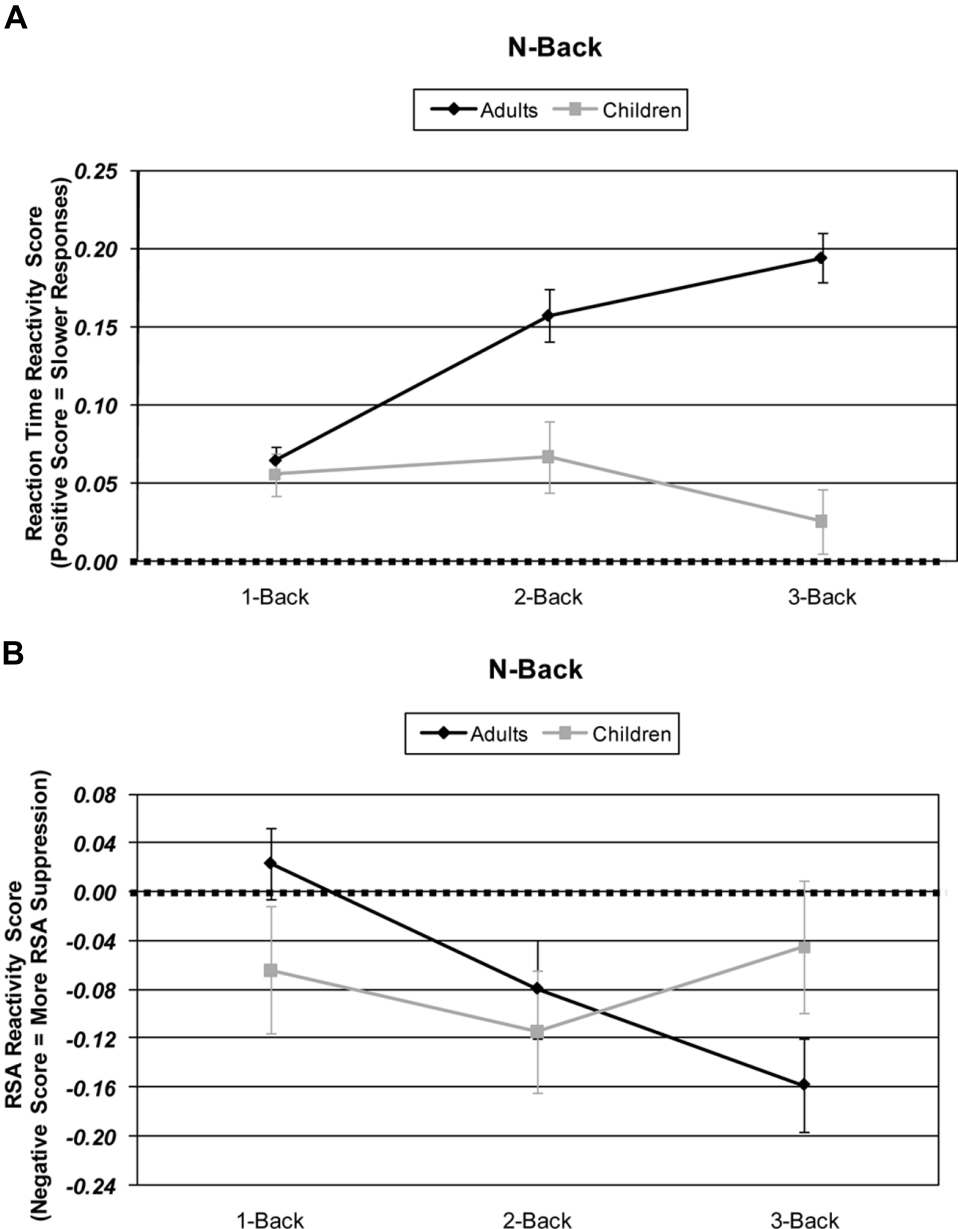
**Age and working memory-load-level differences in: **(A)** N-Back behavioral performance responsitivity and as measured by response time (ms). (B)** N-Back physiological response reactivity as measured by high frequency heart rate variability (HRV). Skew-corrected reactivity for each difficulty level is calculated by subtracting that difficulty condition’s HRV from the 0-back difficulty condition HRV.

An Age × Difficulty Condition (2 × 3) ANOVA of HRV responsitivity revealed no significant main effects of age [*F*(1,57) < 1], but did reveal a main effect of difficulty [*F*(2,114) = 4.57, *p* = 0.014, partial η^2^ = 0.074]. Additionally, adults and children differed in their HRV responsitivity to increasing WM difficulty resulting in a significant age by difficulty interaction [*F*(2,114) = 5.86, *p* = 0.005, partial η^2^ = 0.093]. Adults’ HRV responsitivity was suppressed with each increasing level of WM difficulty [1- vs. 2-back: *t*(33) = 2.96, *p* = 0.006; 2- vs. 3-back: *t*(33) = 2.54, *p* = 0.016; 1- vs. 3-back: *t*(33) = 4.69, *p* < 0.001]. Although the children’s means in the 1- and 2-back conditions appeared reactive to WM load, the children’s HRV responsitivity did not significantly differ among WM difficulty levels (all *t*s < 1.46, *p*s > 0.158). The adults’ and children’s 1-back HRV responsitivity was not significantly different from baseline, and, interestingly, the children’s 3-back HRV responsitivities were not significantly suppressed below baseline [*t*s < 1.24, *p*s > 0.113]. See **Figure 2**.

Because the younger and older children in earlier analyses showed different performance on the 2- and 3-back conditions, each age subgroup was compared to adults. The younger subgroup of children differed in significance compared to the response time of adults for both 2- and 3-back conditions [*t*s(45) > 4.12, *p*s < 0.001]. The older children were not significantly different than adults in suppression of response time for the 2-move condition [*t*(44) = 1.25, *p* = 0.219], but these older children differed in their performance time on the 3-back problems [*t*(44) = 3.84, *p* < 0.001].

## DISCUSSION

The current study builds on past literature by examining developmental differences in HRV responsitivity to increased executive load. Both child and adult groups were assessed across multiple executive function tasks focused on three critical facets of executive functioning. Each task was designed to incrementally increase the executive control necessary for correct and rapid responses, and also assess those executive functions found to be related to executive effort and HRV in the past literature (Thayer et al., 2009). Our task designs were validated by behavioral results. For both age groups and all executive function tasks, behavioral performance was suppressed with increased executive load. Generally, adults were more behaviorally reactive, showing larger decreases in speed of performance with increasing load, as compared to children.

Results for the HRV responding were more complex. The two tasks that required a series of discrete, timed response in relatively rapid succession – the inhibition and WM tasks – produced HRV suppression that was reactive to increased executive cognitive load. It may be that the time pressure of fast responding caused a cognitive state requiring an overwhelming amount of executive control. When HRV suppression was produced it was again more reactive overall and also more reactive to increased load in adults [similar to reduced HRV responsitivity in children during a version of the Stroop task by Mathewson et al. (2010)]. The more complex multistep planning task, which required slow, self-paced responses over a longer time than the other tasks, showed behavioral responsitivity while not producing any significant HRV responsitivity for either age group.

**FIGURE 3:**
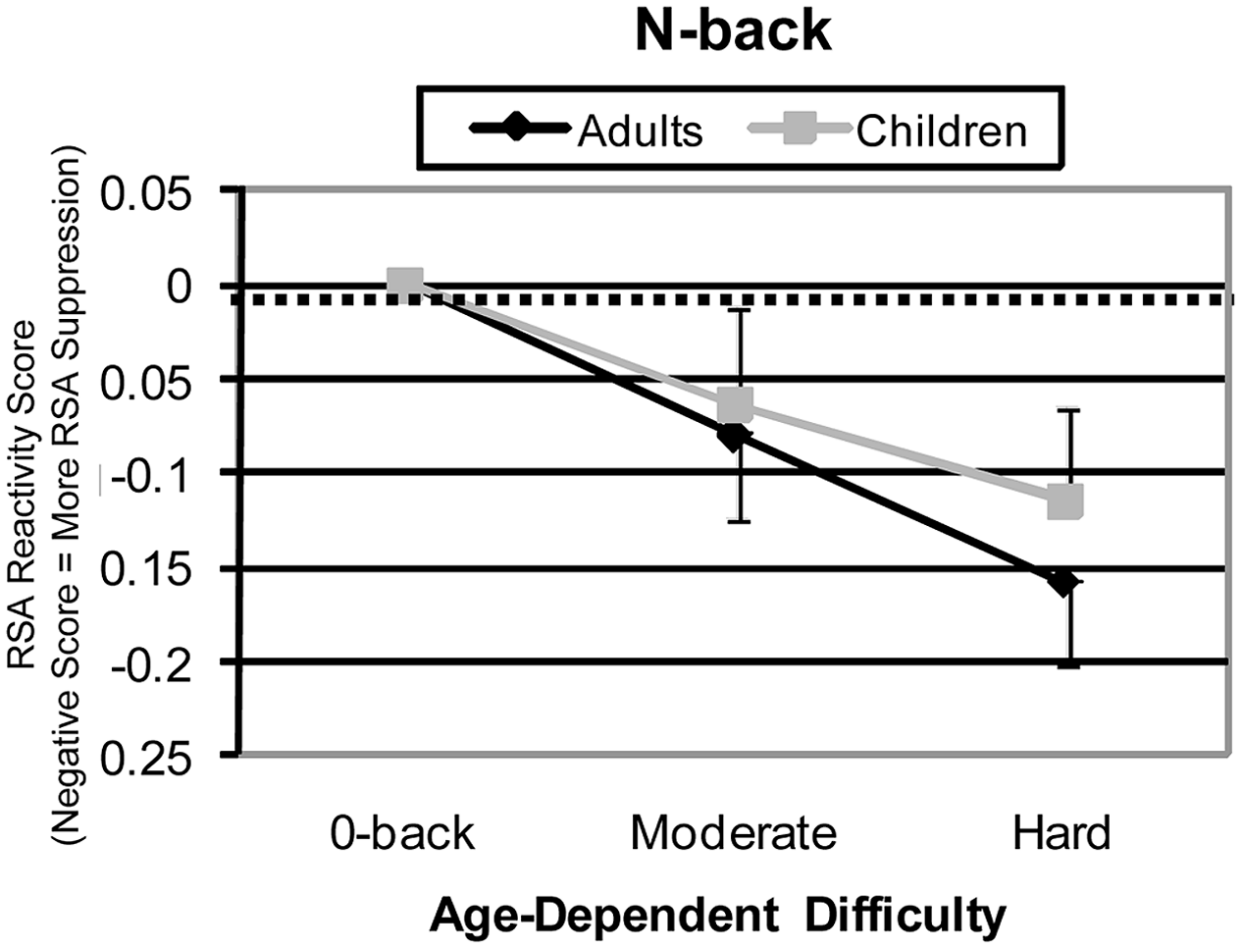
**N-Back high frequency HRV reactivity for effective (age-equated) difficulty levels.** For adults, medium, and hard difficulty conditions were 2-back and 3-back. For children, medium and hard difficulty conditions were 1-back and 2-back conditions. Adjusted patterns are shown for HRV reactivity.

The N-back, with its multiple levels of difficulty across a wide range of WM loads, may offer the most insight into developmental differences in HRV responsitivity to executive load. With this task, it is possible to examine multiple levels of *effective difficulty*, which can also be conceptualized as age-group-specific levels of moderate and high difficulty [an analysis approach suggested by Katsoni et al. (2006)]. The 1- and 2-back conditions can be reasonably viewed as moderate and high effective difficulty levels in children, and 2- and 3-back conditions can be reasonably viewed as moderate and high difficulty levels in adults. With this assignment, a different comparison across age can be assessed. When this age-specific difficulty adjustment is made, similarity rather than difference appears (see **Figure 3**). Specifically, patterns of HRV suppression are similar between age groups. This suggests that when subjective difficulty requires similar amounts of effort, children and adults may show similar effort-related HRV suppression.

Of course, the obvious question with this interpretation is: “What about the 3-back with children – isn’t it also very difficult?” The reason we exclude this condition here is that we interpret the whole of the results, behavioral as well as HRV, as an indication that the children appeared to be overwhelmed by the most demanding, 3-back condition of the N-back task. The strongest evidence of this was that behavioral performance was near chance. The children may have given up mental effort during this most difficult condition. The result to be expected, if this is the case, is little HRV suppression, just what we found.

### HRV AS AN INDEX OF EXECUTIVE EFFORT

Adults’ HRV responsitivity increased with increased executive loads in the inhibition and WM tasks, but not the planning task. These patterns suggest that HRV does index some forms of executive effort, perhaps those that require assessing a rapid series of discrete stimuli while processing and responding in a speeded manner with a relatively high density of responses, similar to those tasks used in past studies of HRV-Executive Function relationships (see Thayer et al., 2009 for a review). Speeded and high density responses were characteristics of our inhibition and WM tasks. Slower, self paced, and multi step responses required by our planning task may require a form of executive functioning not indexed by HRV. This implies that HRV suppression is sensitive to a specific form of attentional control requiring vigilance to a rapidly change course of stimuli not under the participant’s control rather than a largely stationary stimulus where responding is under the participant’s control. An alternative administration of a planning task with more rapid presentation of problems and a single button response would be more similar to our inhibition and WM tasks and would allow us to determine further if planning is an executive function reflected in HRV responsitivity. We also could have offered a simpler planning baseline, such as 1- and 2-move problems, and then perhaps we would have seen planning difficulty differences. Finally, this data was collected before Berg et al. (2010) as well as Kaller et al. (2004) published other problem parameters other than minimum number of moves that determine difficulty. Not controlling for these other parameters may have created noise and overlap between out TOL conditions preventing a clear parametric design for this task.

The large amount of children’s HRV variance during the planning task may have resulted from the multiple slow responses for a single solution and from variance in the strategy/approach to the task. For example, the current study’s instructions and reward schedule encouraged planfulness, but the Tower of London task, like other tower-transfer planning tasks, can be approached with strategies requiring more or less multistep planning. Participants may use lower planning effort strategies that still reach the goal using strategies based on surface appearance and making random moves hoping to “chance upon” the solution (Berg et al., 2006). During the most difficult planning task conditions, child participants may have been switching among approaches requiring more and less executive effort, with some moves or sequences of moves during the solution period being more planful than others. There is some evidence in the data to support that children were varying more greatly in switching among different, more and less effortful approaches or strategies when faced with the most difficult planning load. This variability was larger in the most difficult Tower of London condition (0.134) than in the most difficult conditions of the Stroop (0.115) and N-back (0.117) tasks. This pattern of variances was not present in the adults (0.070, 0.063, and 0.062 relatively).

This interpretation of HRV’s sensitivity to strategy also matches well with the pattern of behavioral and HRV responsitivity that children displayed during the most difficult condition in the WM task. When overwhelmed with the most difficult, 3-back condition of the WM task, the children appear to have switched to a less executive/effortful strategy for this task, perhaps responding based on familiarity rather than encoding each item (Speer et al., 2003).

### ADDRESSING HYPOTHESES

We hypothesized that incremental increases in executive load would result in incremental decreases in behavioral performances and HRV. This pattern was present in the adults during the WM task, showing incrementally more suppressed HRV along with incrementally poorer performance. This incremental HRV change may be most evident in the task that had many (4) levels of difficulty and which required vigilance and speeded responding to rapidly presented stimuli. Except for the most difficult condition, where children were overwhelmed, children’s responses were also incremental in appearance.

### CLINICAL SIGNIFICANCE OF FINDINGS

There are some clinical ramifications of the current study, specifically when clinicians are determining test design to monitor what executive functions may be at deficit. Those that use a more time pressure, speeded response may tax a different form of executive functioning than a task that is self-paced. Developmentally, this study underscores the importance of choosing age-appropriate difficulty levels of executive functioning tasks, as the giving-up behavior in the most difficult N-back condition, poor performance can occur not because the participant is trying and struggling, but simply because they are giving up.

### LIMITATIONS OF CURRENT RESEARCH AND FUTURE DIRECTIONS

The most serious limitations for this study come from the planning task, where there were no high frequency phasic HRV differences found with increasing executive load. We hesitate to think that planning as an executive function is not indexed by HRV, but think that the way that we administered the planning task may have limited the HRV responsitivity. One potential design aspect that could have hidden HRV responsitivity is that difficulty levels were not spread far enough among easy, medium, and difficult conditions. Future studies may wish to vary planning difficulty as widely as WM difficulty, with baselines of 1 or 2 move problems, and difficulties ranged widely as it was between 0-back and 3-back. With this change in design we could compare very low planning load, moderate planning load, and high planning load. This may show one of the limitations of the parametric design, that a full range of difficulty must be presented.

Additionally, the pattern in the results where we saw HRV responsitivity in WM and inhibition executive functioning tasks, but not the planning task may have also revealed that high frequency phasic HRV is most sensitive to increases in executive function load when there is some time pressure in response, as there was in our Day-Night Stroop and N-back tasks. Perhaps we would have seen a planning difference if we had told the participants to solve as quickly as possible, or perhaps if we had given them a different variation on the Tower of London, one more similar to how it is used in fMRI studies where participants see the start and goal positions, solve problems covertly, in their mind’s eye, and then respond either with a button press of how many moves it take or solving the problem with mouse movements (Unterrainer et al., 2004b).

This idea of speeded responding being more strongly indexed by HRV may relate to one of the other applications of HRV, to emotional regulation (Thayer and Lane, 2000) and specifically to anxiety (Appelhans and Luecken, 2008). It may be that the Stroop and N-back with their speeded responding were more anxiety provoking, than the planful moves approach that was the best approach for the TOL. The more difficult Stroop and N-back conditions may have caused more anxiety or emotional dysregulation than easier conditions, while with the TOL solving fewer moves did not cause less emotional dysregulation than more difficult conditions. This again points to future studies putting executive functions on an even field as to speeded response, with TOL having to be solved in the head as quickly as possible.

Our use of performance based reward, which participants did not see until the end of the task, may have also played a role in which executive tasks showed HRV responsitivity. In past literature, reward have been seen to make a difference in the performance of certain, gambling-related executive functioning tasks, that is for reward for a different odds-based game of chance context with preschoolers (Kerr and Zelazo, 2004). Concerns of the reviewers suggest that future studies should be conducted to determine if reward, such as offered in our study change the anxiety level in the certain, time pressured tasks.

## CONCLUSION

In sum, high frequency phasic HRV appears sensitive to increasing executive demand in adults and children for WM and inhibition tasks. The exception to this was in the WM condition that was too difficult for the children, where there performance reverted to chance levels, suggesting the children were just guessing responses, and their HRV returned closer to baseline. We were most surprised by the findings with the planning task, where there was no HRV responsitivity with increased planning load. We discussed above why that may be so, and how future studies can investigate if planning is truly an executive function that does not have an impact on HRV or if HRV is sensitive to some of the task parameters that a multi-step planning task may have, as compared to a simple, single button/single click time-pressured task, such as our N-back and Day-Night Stroop tasks.

The children’s HRV was less reactive than adults suggesting that decreased frontal lobe involvement in these children may impact the sympathetic and parasympathetic systems such that there is decreased HRV responsitivity. This is somewhat surprising, as children’s time locked evoked heart rate responses are larger than adults, children’s HRV could have been more reactive (Byrd and Berg, 2002).

## Conflict of Interest Statement

The authors declare that the research was conducted in the absence of any commercial or financial relationships that could be construed as a potential conflict of interest.

## Footnotes

1 An Age × Task × Order ANOVA revealed no significant Order × Task or Age × Order × Task interactions on any of the performance measures (*p*s > 0.05).

2 Respiration was recorded contemporaneously with the heart rate using a 10 cm mercury strain gage attached to an elastic belt wrapped snuggly around the participants’ chests. The gage was attached to a Parks Electronics Model 270 plethysmograph transducer box to convert the signal to recordable voltages. Respiration data was sampled by a Tecmar 12 bit A/D converter at a rate of 10 Hz for the 30-s epoch and, using DOS-based custom software, recorded onto a computer hard disk. An FFT was used to determine the power of the frequencies present within this respiration signal.
